# Gene Expression Behavior of a Set of Genes in Platelet and Tissue Samples from Patients with Breast Cancer

**DOI:** 10.3390/ijms24098348

**Published:** 2023-05-06

**Authors:** Luis A. Burciaga-Hernandez, Cecilia F. Cueto-Villalobos, Nancy Ortega-Piñon, Irma E. Gonzalez-Curiel, Susana Godina-Gonzalez, Gwendolyne Mendez-Frausto, Anna P. Aguilar-Esquivel, Vilma Maldonado-Lagunas, Luis E. Guerrero-de la Torre, Jorge Melendez-Zajgla, Erika K. Sanchez-Garcia, Irma B. Mitre-Aguilar, Gretel Mendoza-Almanza

**Affiliations:** 1 Maestría en Ciencias Biomédicas, Universidad Autónoma de Zacatecas, Zacatecas 98160, Mexico; luis.burciaga@uaz.edu.mx (L.A.B.-H.);; 2 Unidad Académica de Ciencias Biológicas, Universidad Autónoma de Zacatecas, Zacatecas 98068, Mexico; 35165676@uaz.edu.mx; 3 Unidad Académica de Ciencias Químicas, Universidad Autónoma de Zacatecas, Zacatecas 98160, Mexico; 36170554@uaz.edu.mx; 4 Laboratorio de InmunotoxicologÍa y Terapéutica Experimental, Unidad Académica de Ciencias QuÍmicas, Universidad Autónoma de Zacatecas, Zacatecas 98160, Mexico; irmacuriel@uaz.edu.mx (I.E.G.-C.);; 5 Laboratorio de Biomarcadores, Unidad Académica de Ciencias QuÍmicas, Universidad Autónoma de Zacatecas, Zacatecas 98160, Mexico; 6 Hospital General Zacatecas “Luz González Cosío”, Zacatecas 98160, Mexico; 7 Laboratorio de Epigenetica, Instituto Nacional de Medicina Genomica (INMEGEN), Ciudad de México 14610, Mexico; 8 Laboratorio de Genomica Funcional del Cancer, Instituto Nacional de Medicina Genomica (INMEGEN), Ciudad de México 14610, Mexico; 9 Unidad de Bioquímica, Instituto Nacional de Ciencias Médicas y Nutrición Salvador Zubiran (INCMNSZ), Ciudad de México 14080, Mexico; 10 Consejo Nacional de Ciencia y Tecnología, Ciudad de México 03940, Mexico

**Keywords:** variation in mRNA levels, gene overexpression, breast cancer, biomarkers panel, tumor-educated platelets

## Abstract

The tumor microenvironment (TME) is constituted by a great diversity of highly dynamic cell populations, each of which contributes ligands, receptors, soluble proteins, mRNAs, and miRNAs, in order to regulate cellular activities within the TME and even promote processes such as angiogenesis or metastasis. Intravasated platelets (PLT) undergo changes in the TME that convert them into tumor-educated platelets (TEP), which supports the development of cancer, angiogenesis, and metastasis through the degranulation and release of biomolecules. Several authors have reported that the deregulation of *PF4*, *VEGF*, *PDGF*, *ANG-1*, *WASF3*, *LAPTM4B*, *TPM3*, and *TAC1* genes participates in breast cancer progression, angiogenesis, and metastasis. The present work aimed to analyze the expression levels of this set of genes in tumor tissues and platelets derived from breast cancer patients by reverse transcription-quantitative polymerase chain reaction (RTqPCR) assays, in order to determine if there was an expression correlation between these sources and to take advantage of the new information to be used in possible diagnosis by liquid biopsy. Data from these assays showed that platelets and breast cancer tumors present similar expression levels of a subset of these genes’ mRNAs, depending on the molecular subtype, comorbidities, and metastasis presence.

## 1. Introduction

Platelets are small (3 to 4 µm) anucleate cells derived from megakaryocytes [1], which have some organelles, including mitochondria, ribosomes, alpha granules, dense granules, and lysosomes [2]. In addition, it is now known that platelets have a functional spliceosome that processes premature mRNA into mature mRNA molecules, pre-miRNA to miRNAs, and presents mRNA translation [3]. Thanks to these organelles and machinery, platelets can regulate gene expression even without containing a nucleus [4], in addition to their widely recognized and documented activities related to hemostasis, vascular integrity, defense against pathogens, and inflammatory events [5].

In recent decades, the role played by platelets in cancer development and metastasis has gained significant relevance. One of the most exciting characteristics of platelets is their ability to obtain feedback from the “disease microenvironments” (DME) into which they enter, releasing biomolecules and internalizing others. Biomolecules taken from the DME are transported to distant sites, contributing to the development and progression of various pathologies [6], such as cancer [7] or COVID-19 [8]. This material exchange can be carried out in various ways, such as direct contact between platelets and damaged cells [9] or through acquisition of platelet-derived microvesicles or cellular-derived microvesicles [10]. In the TME, platelets show great activity and communication with fibroblasts, endothelial cells, inflammatory cells, cancer-associated fibroblasts, mesenchymal stromal cells [10], tumor-associated macrophages, and cancer stem cells. Platelets enrich the TME with a great diversity of biomolecules, such as cytokines, growth factors (GF) [11], molecules anchored on the cell membrane’s outer surface [12,13], secretion proteins, mRNAs, and miRNAs [12].

Platelets and tumor cells educate each other. Thus, it is known that in addition to TEP, there are platelet-educated tumor cells (PET) [13]. In recent years, several studies on different types of cancer, based on massive mRNA sequencing techniques, have shown changes in the mRNA profiles of platelets from cancer patients compared to the mRNA profiles of platelets from healthy individuals. This fact could indicate that platelets are an excellent, non-invasive biological marker for the diagnosis and prognosis of cancer since those studies managed to distinguish between diseased and healthy individuals, and even between the early and advanced stages of the disease [14,15,16,17,18,19,20,21].

These findings suggest that platelets have great potential as diagnostic markers for different types of cancer. They can also detect it in the early stages of development. However, most studies on the subject require validation in different populations and larger samples.

According to data from the World Health Organization for 2020, breast cancer continues to be the most recurrent and deadly cancer in women worldwide [22]. It is a heterogeneous, highly diverse disease that has been classified according to some common characteristics associated with the evolution of the disease, prognosis, and treatment, such as histology and the state of membrane receptors [23]. An early and selective diagnosis of the different molecular subtypes of cancer is essential to select the most appropriate treatment.

The genes *PF4* [24], *VEGF* [25], *PDGF* [26], *ANG-1* [27], *WASF3* [28], *LAPTM4B* [29], *TPM3* [9], and *TAC1* [30] have been separately evaluated at some point in samples of breast tumor tissue or platelets from breast cancer patients. All of them are characterized by presenting an overexpression in the case of those analyzed in tissue (*VEGF*, *PDGF*, *ANG1*, *LAPTM4B*, *WASF3*) or an increase in mRNA levels in the case of those reported in platelets (*TPM3*, *PF4*, *TAC1*). In the present work, we evaluated the expression of these genes in the molecular subtypes of breast cancer, both in platelets and breast tumor tissues, by RT-qPCR to conclude if some of these markers could work together to detect the molecular subtype of breast cancer through a liquid biopsy.

All the analyzed genes had variations in mRNA levels or overexpression in platelets and tumor tissues, respectively, as compared to samples from healthy volunteers and control tissues. We found a panel of genes with statistically significant fold-change values in platelets and tumor tissue in each molecular subtype of breast cancer.

## 2. Results

### 2.1. Characteristics of Patients and Samples

Table 1 summarizes the characteristics of the patients and healthy volunteers who donated platelets. The age ranges between healthy donors and breast cancer patients were found to be statistically different. However, a considerable number of patient and control samples shared the same age range (Supplementary Figure S1).

After isolating platelets from whole blood, a quality test was randomly performed in a subset of the samples. A smear of purified and concentrated platelets was stained according to the Wright technique. A total of 30 randomized tests were performed. Supplementary Figure S2 shows an example of the purity of the isolated platelets. Flow cytometry analyzed a triplicate sample of each step in the purification of platelets to observe the groups formed according to the size. The purified platelets were labeled with CD41 to prove that the isolated cell subpopulation corresponded solely to platelets (Supplementary Figure S3).

To analyze the impact of age on the variation in mRNA levels of platelets from breast cancer patients, we stratified patients by age (31–40, 41–50, 51–60, 61–70) to determine whether the observed changes in mRNA levels were due to age in this group of people or to their disease. According to the statistical analysis, there was no significant difference in gene expression between breast cancer patients based on their age (Supplementary Figure S4). The changes were dependent on the presence of cancer.

Table 2 summarizes the characteristics of formalin-fixed paraffin-embedded (FFPE) breast tissue donor patients.

### 2.2. Gene Expression in Platelets and Tumor Tissue from Patients with Breast Cancer

**Figure 1:**
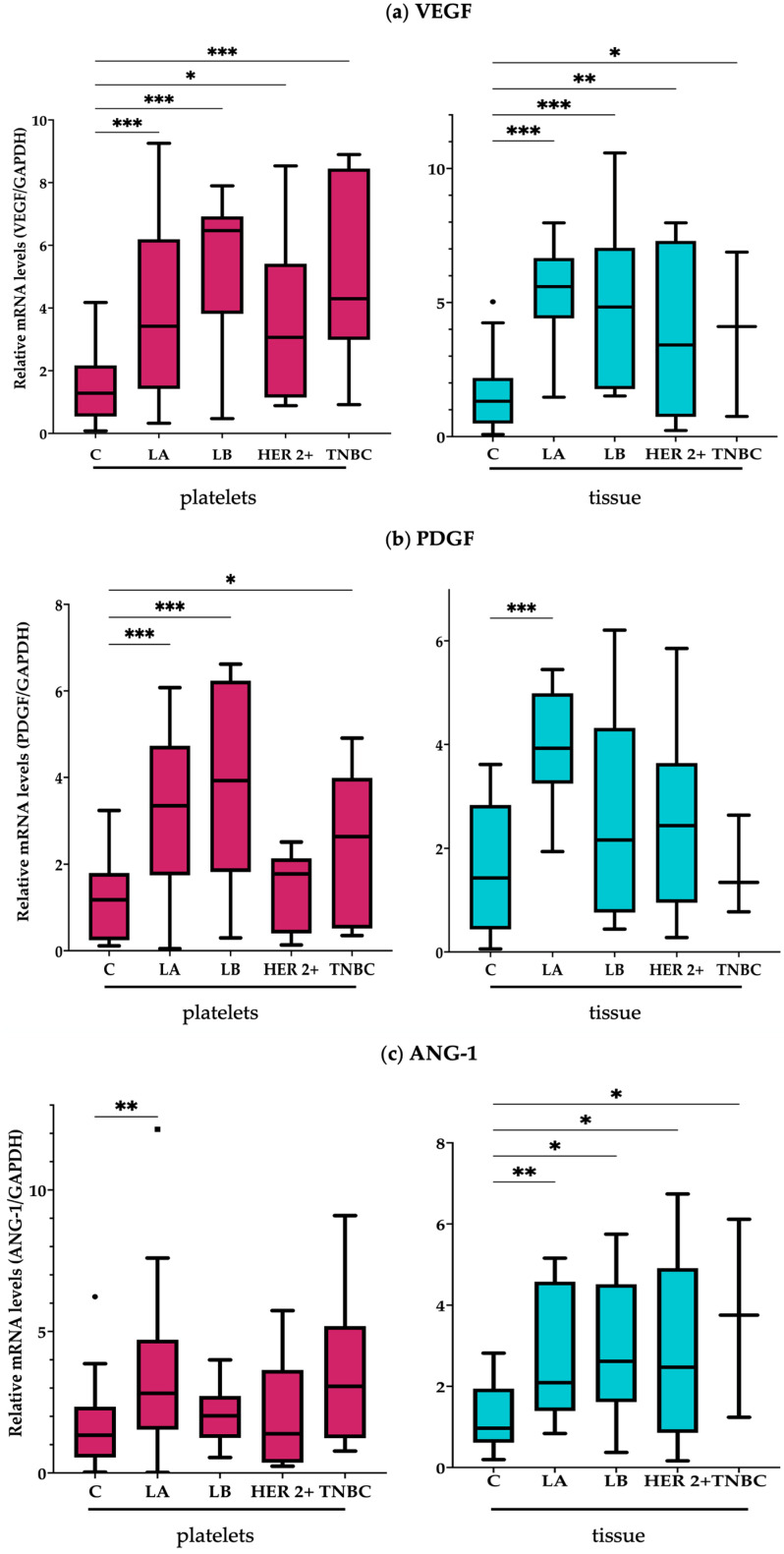

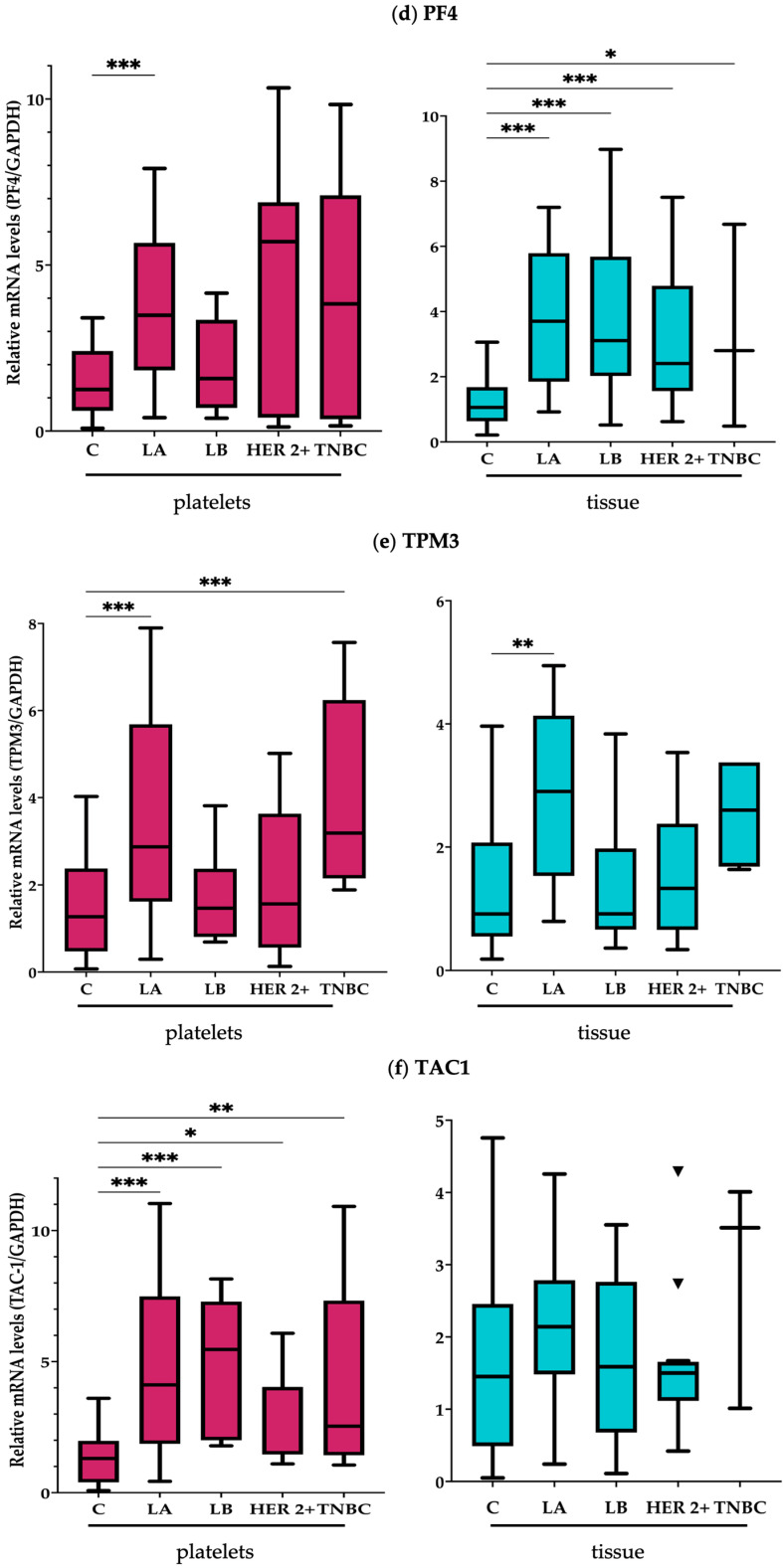

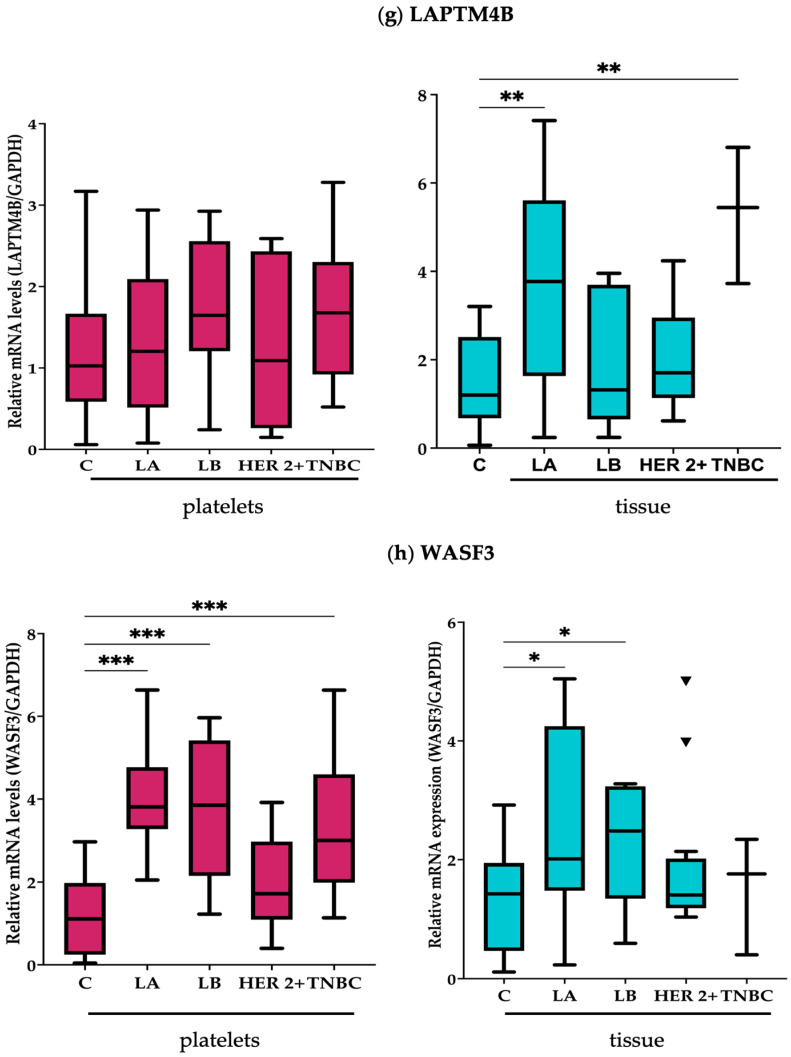
Increased expression of tumor-related genes in platelets and breast tumoral samples from cancer patients. RT-qPCR analysis of genes related to breast cancer development, angiogenesis, and metastasis from platelets (**left**) and breast tissues (**right**), derived from breast cancer patients and healthy donors. Box plots depict the expression fold differences of the eight analyzed genes between healthy donors and breast cancer patients. The statistical significance was denoted by *, **, ***, representing *p*-values of ≤0.05, ≤0.01, ≤0.001, respectively, by the Kruskal–Wallis test. C: control; LA: luminal A tumors; LB: luminal B tumors; TNBC: triple-negative breast cancer.

To assess if the platelet expression of a panel of breast cancer-associated genes could be similar to the breast tumor, we measured the expression levels of these genes in RNA derived from platelets and tumor tissue from patients with breast cancer and control groups (without malignant neoplasms). The set of selected genes was composed of a group of angiogenesis-related genes (*VEGF*, *PDGF*, *ANG-1*, and *PF-4*) and genes that have been previously associated with carcinogenic or metastatic processes in breast cancer (*WASF3*, *LAPTM4B*, *TPM3*, and *TAC1*). As expected, we found that the expression of the selected genes differed between breast tissue samples from cancer patients and healthy donors. Interestingly, we found that these genes were also upregulated in platelets derived from cancer patients (Figure 1). Expression of these genes differed among the molecular subtypes of breast cancer (luminal A, luminal B, HER2+, triple-negative breast cancer (TNBC)), as not all molecular subtypes have the same expression profile (Figure 1). In the luminal A subtype, the variation in mRNA levels and overexpression of the *VEGF*, *PDGF*, *ANG1*, *PF4*, *WASF3*, and *TPM3* genes coincided in both types of samples (platelets and tumor tissue, respectively) (Figure 1a–e,h). In luminal B, the variation in mRNA levels and overexpression of *VEGF* and *WASF3* coincided in both types of samples (Figure 1a,h). HER2+ was only found to be variation in mRNA levels and overexpressing *VEGF* in both types of samples (Figure 1a). Variation in mRNA levels and overexpression of TNBC, *VEGF*, and *ANG-1* coincided in both samples (Figure 1a,c). Additionally, we found differences in the types of genes that were only variate in mRNA levels or overexpression in one type of biological sample in one molecular subtype. In the four molecular subtypes, the *TAC1* gene exclusively had variation in mRNA levels in platelets from patients with breast cancer (Figure 1f). Furthermore, the *WASF3* and *TPM3* genes were found to show variation in mRNA levels only in the TNBC molecular subtype (Figure 1e,h). The *LAPTM4B* gene was overexpressed in tumor tissues of the luminal A, luminal B, and TNBC subtypes (Figure 1g). Tumors with the HER2+ molecular subtype expressed *ANG-1* and *PF4* (Figure 1c,d). Genes related to angiogenesis processes, such as *VEGF*, *PDGF*, *ANG-1*, and *PF-4*, were differentially expressed in luminal A in breast cancer tissue and in platelets from patients with BC (Figure 1a–d).

**Figure 2:**
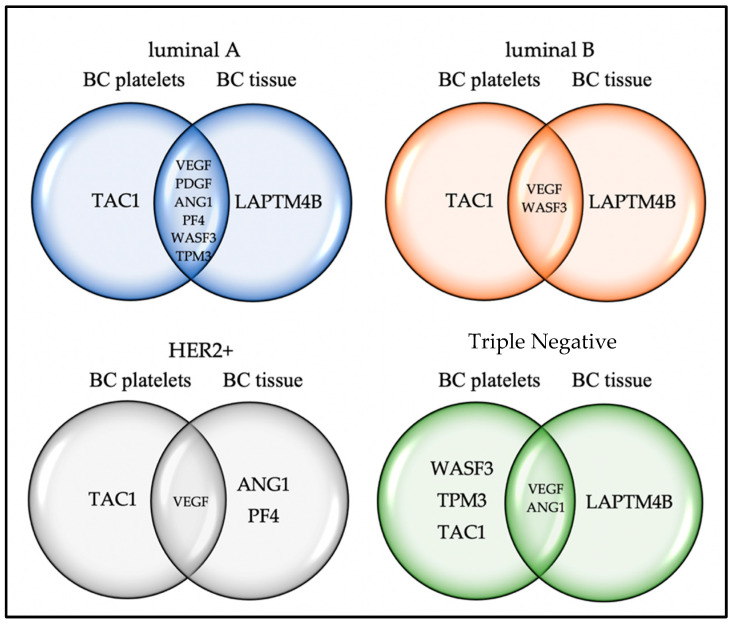
Summary of the profile of differentially expressed genes in both types of biological samples (platelets and tissues) between a group of healthy donors and patients with different subtypes of breast cancer. In the four molecular subtypes, the *TAC1* gene exclusively had variation in mRNA levels in platelets from patients with breast cancer. Furthermore, only in the TNBC molecular subtype did the *WASF3* and *TPM3* genes have variation in mRNA levels. The *LAPTM4B* gene was found to be overexpressed in tumor tissue of the luminal A, luminal B, and TNBC cancer subtypes. Tumors with the HER2+ molecular subtype expressed *ANG-1* and *PF4*.

The gene expression results in each of the biological samples analyzed are summarized in Figure 2. We can see that each breast cancer molecular subtype presented a different expression of genes, which would tell us about a characteristic molecular signature in each subtype that could be used to specifically detect the subtype of breast cancer.

**Figure 3:**
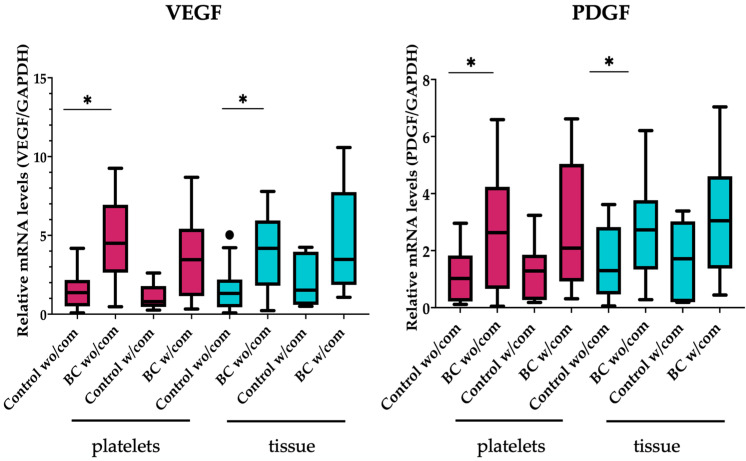

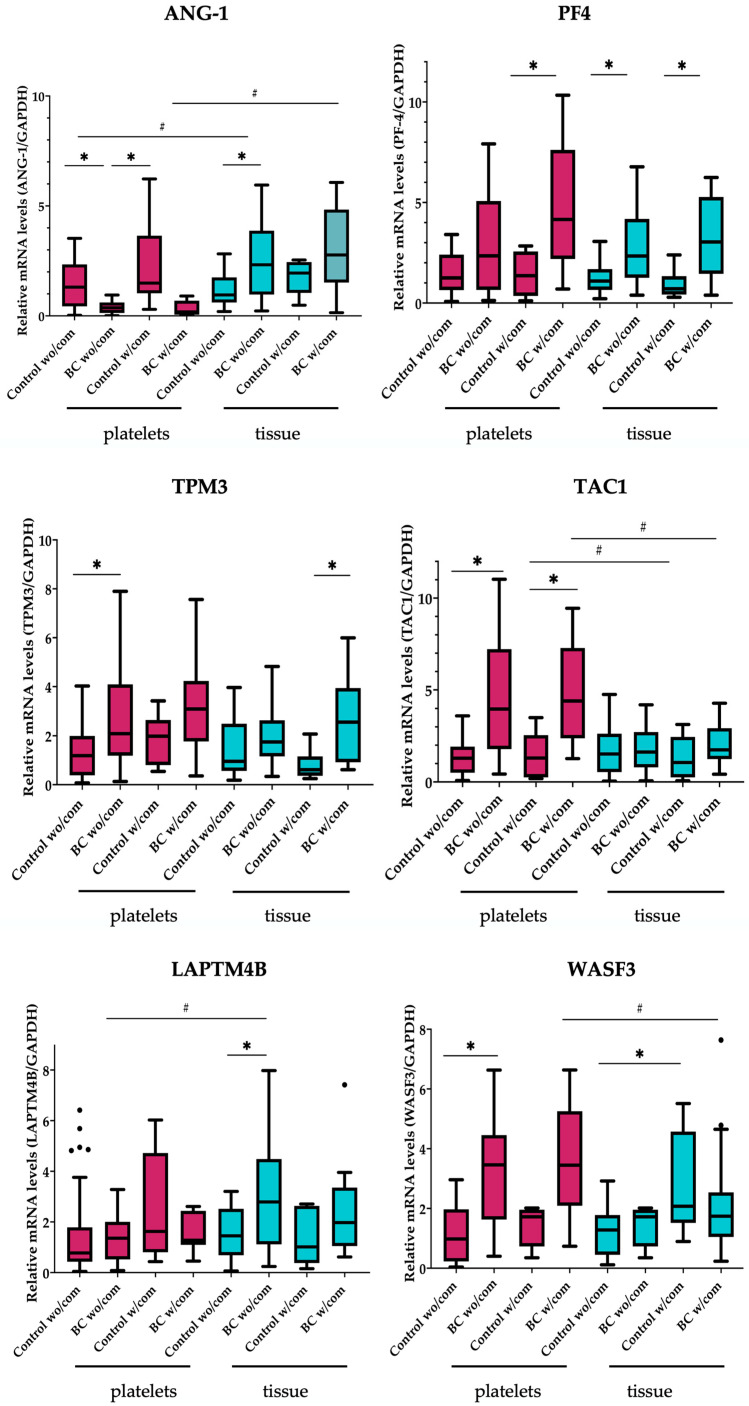
Increased expression of tumor-related genes in platelets and breast tumoral samples from cancer patients. RT-qPCR analysis of genes related to breast cancer development, angiogenesis, and metastasis from platelets (**left**) and breast tissues (**right**), derived from breast cancer patients and healthy donors. Box plots depict the expression fold differences of the eight genes between the healthy donors and breast cancer patients, stratified by comorbidities. The statistical significance was denoted by *p*-values ≤ 0.05 (*), and comparisons were performed within the same tissue sample (platelets or tissue) or between groups of different origin (platelets and tissue) (#) using the Kruskal–Wallis test. wo/com: Without comorbidity; w/com: with comorbidity.

**Figure 4:**
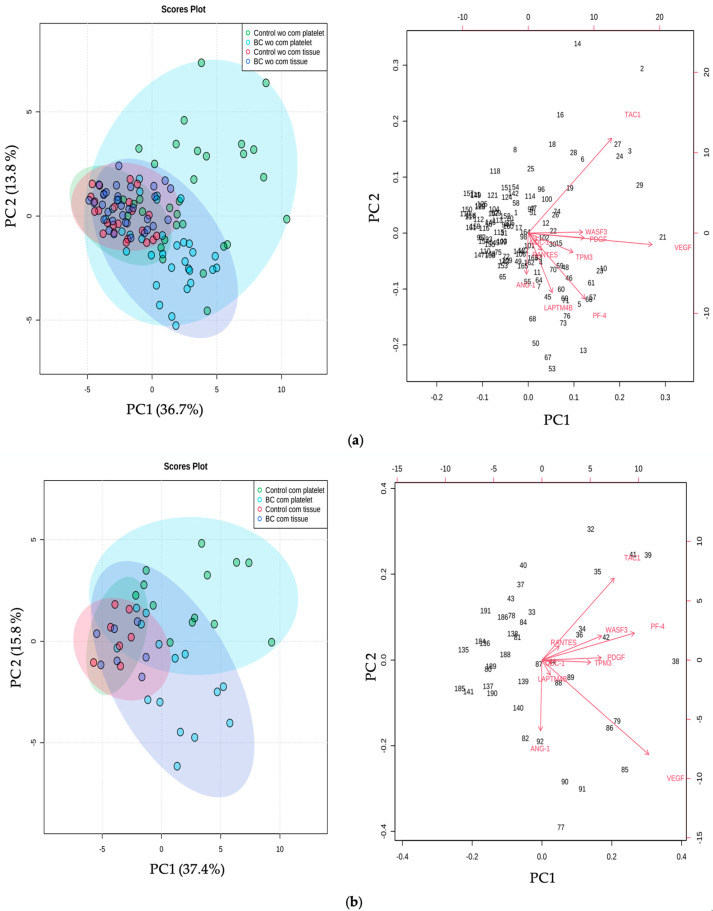
Principal component analysis (PCA). The PCA shows the clustering pattern of the analyzed genes in both types of samples, platelets and breast tissue, from breast cancer patients and healthy donors, stratified as (**a**) without comorbidities and (**b**) with comorbidities. The explained amount of the total variance of the entire dataset is shown for each principal component, PC1 and PC2. wo/com: Without comorbidity; w/com: with comorbidity.

Subsequently, we analyzed factors affecting gene expression in platelets, such as the most common comorbidities. Comorbidity is defined as the simultaneous presence of two or more disorders or pathologies in a person. In the Mexican population, hypertension and diabetes are the two main comorbidities associated with a large number of chronic diseases, such as cancer, for which it was essential to analyze changes in gene expression and mRNA levels based on the presence of comorbidities in patients and control groups, as shown in Figure 3 and Figure 4.

Figure 3 and Figure 4 show comorbidities’ association with gene expression in patients with breast cancer. We observed that the presence or absence of other diseases influences gene expression (tissue) and mRNA levels (platelets). In order to analyze the influence on gene expression in breast cancer patients of the presence or absence of comorbidities, we grouped both tissue and platelets based on this parameter. *VEGF*, *PDGF*, and *ANG-1* gene expression levels were increased in tissue and platelets when no comorbidities existed. *TPM3*, *TAC1*, and *WASF3* significantly increased mRNA levels without comorbidities in platelets. Only in tumor tissue were *PF4* and *LAPTM4B* significantly increased without comorbidities.

In the presence of comorbidities, *TAC1* and *PF4* were significantly increased in platelets. Moreover, in tumor tissue from patients with comorbidities, there was an increase in the gene expression of *TPM3* and *PF4.*

In tumor tissue, an increased expression of *VEGF*, *PFGF*, *ANG-1*, *PF-4*, and *LAMPTB4* was observed in the group of cancer patients without comorbidities, compared to healthy individuals without comorbidities. The expression of *PF-4* and *TPM3* was increased in cancer patients as compared to control individuals in the tumor tissue of the group of patients with comorbidities.

We also evaluated whether there were any differences in the expression of these transcripts between platelets and tumor tissue from cancer patients with and without comorbidities. In patients without comorbidities, an increase in the expression of *ANG-1* and *LAMPTB4* was observed, while the expression of *TAC-1* was decreased in tumor tissue compared to platelets. Regarding the group with comorbidities, only the expression of *ANG-1* was increased, pointing toward a non-cancer role of this gene. The relationship between the genes under study and comorbidities in samples from tumor tissue or platelets was evaluated using principal component analysis (PCA). The groups without comorbidities analyzed in platelets were separated between healthy individuals and patients with cancer. No such separation was performed in the groups in which the analysis was carried out in tissue tumors (36.7% and 13.8% of the variance, respectively). The variables that best defined the variance were *VEGF*, *TAC-1*, *PF-4*, and *LAPTMB4*. In patients with comorbidities, the *ANG-1*, *VEGF*, *PF-4*, and *TAC-1* genes clustered more closely in tumor tissue than in platelets (37.4% and 15.8% of the variance of PC1 and PC2).

**Figure 5:**
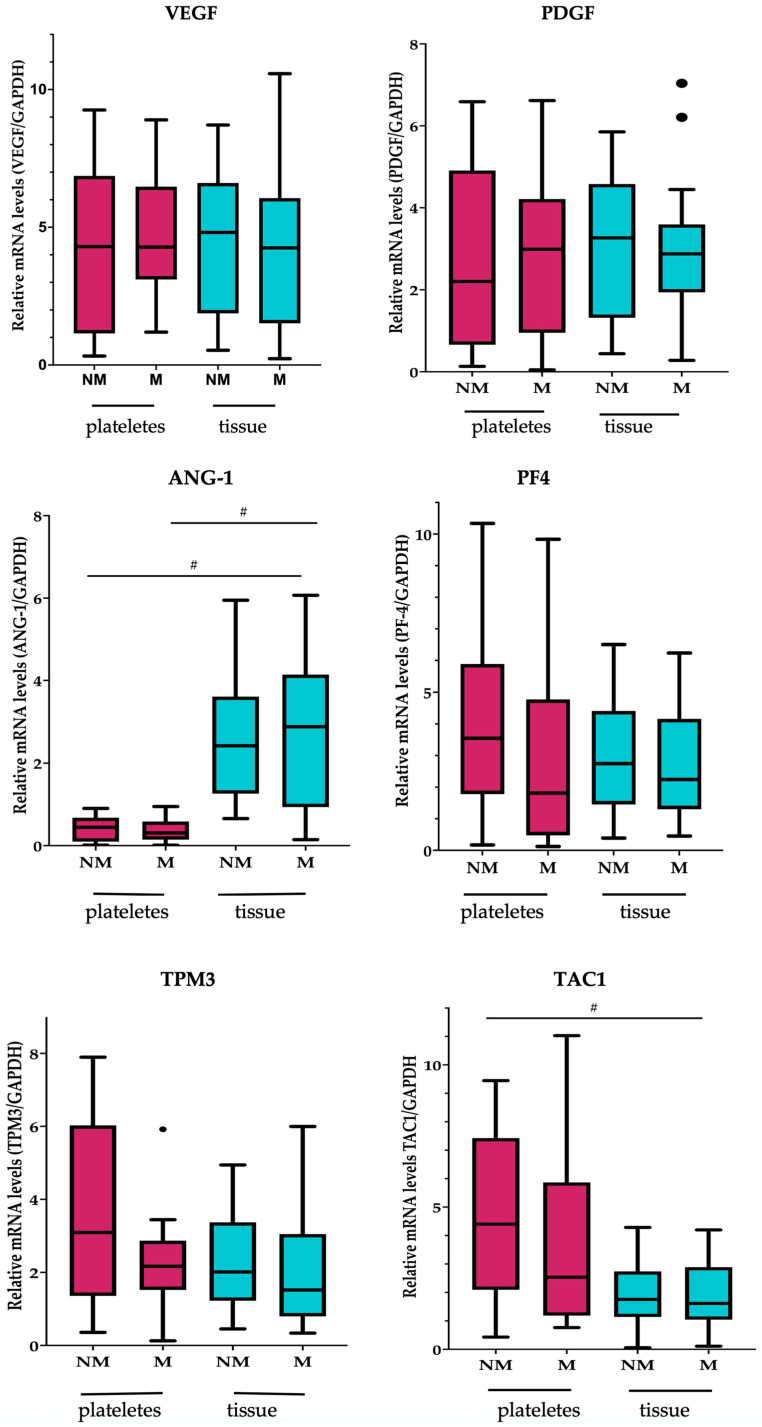

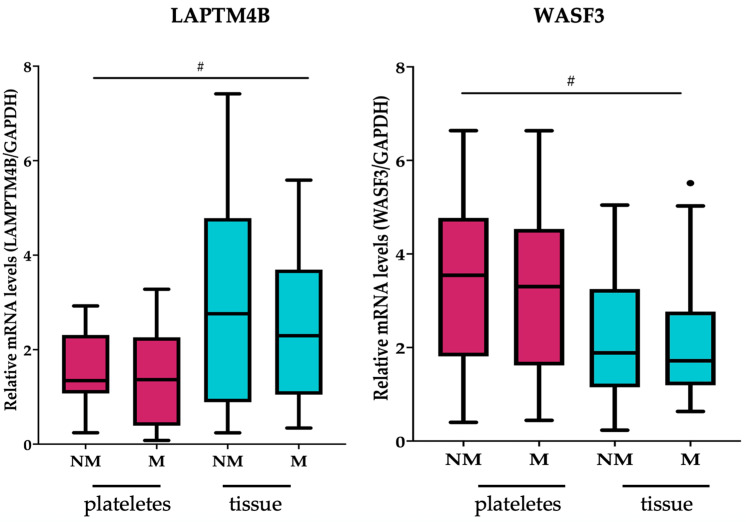
Increased expression of metastasis-related genes in platelets and breast tumoral samples from cancer patients. RT-qPCR analysis of genes related to metastasis in platelets and breast tissue, from breast cancer patients and healthy donors. Box plots depict the expression fold differences of the eight genes analyzed. The statistical significance between groups of different origin (platelets and tissue) was determined by the Kruskal–Wallis test and denoted by *p*-values ≤ 0.05 (#). NM: no metastasis; M: metastasis.

**Figure 6:**
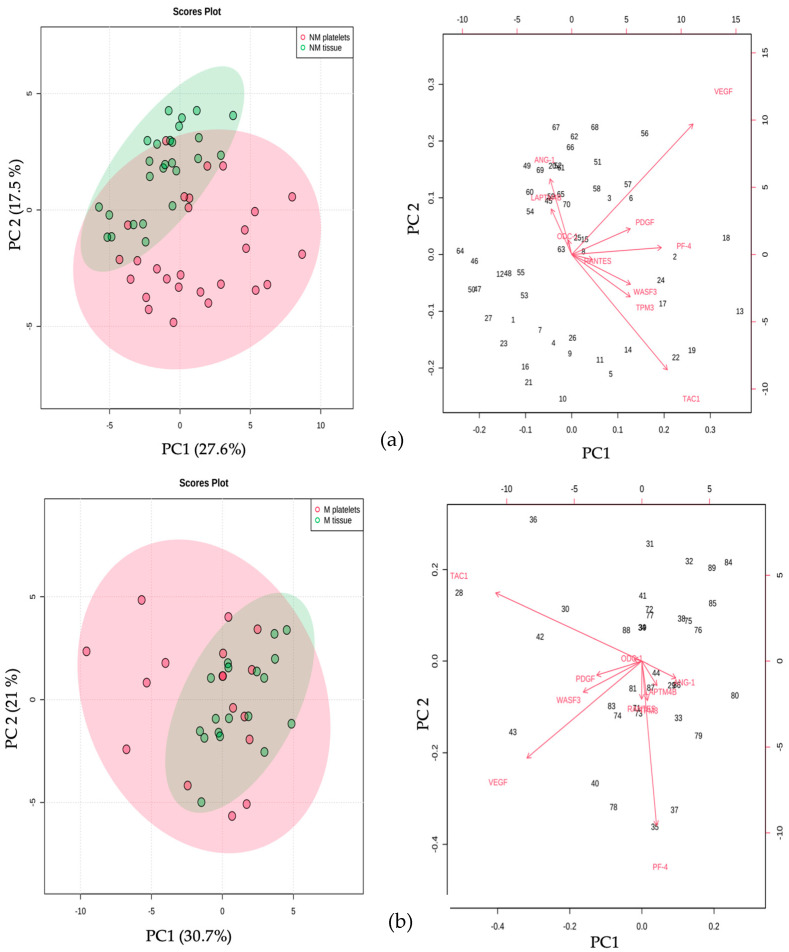
Principal component analysis (PCA). The PCA shows the clustering pattern of the genes analyzed in both types of samples, platelets and breast tissue, from cancer patients, stratified as (**a**) no metastasis and (**b**) metastasis. The explained amount of the total variance of the entire dataset is shown for each principal component, PC1 and PC2. NM: no metastasis; M: metastasis.

Regarding the analysis of the expression of the panel of genes under study in patients with metastasis, it was observed (Figure 5 and Figure 6) that in the group of patients with BC without metastasis, the expression of *ANG-1* and *LAMPT* was increased in the tumor tissue compared to platelets. At the same time, *TAC-1* and *WASF3* were found to be decreased in platelets. In the metastasis group, only *ANG-1* was observed to increase in tumor tissue compared to platelets (Figure 5). In the patients without metastasis, the PCA showed two well-differentiated groups between platelets and tumor tissue. The proportion of variance explained by PC1 was 27.6%, and the proportion explained by PC2 was 17.5%, with *VEGF* and *TAC-1* being the variables that contributed the most to the variance (Figure 6a). Regarding the metastasis group, there was a slight separation between the groups, with 30.7% of the variance explained by PC1 and 21.4% by PC2. The variables that contributed the most to this difference were *VEGF*, *TAC-1*, and *PF-4* (Figure 6b).

Gene expression was compared between cancer patients (without any stratification) and healthy individuals, stratifying between those with comorbidities and those without.

The obtained results indicate that not all genes were affected in their expression by the presence of comorbidities, including the gene expression of platelets.

## 3. Discussion

Platelet number has been used for several years as an associated marker for cancer prognosis. It is known that the physiological range of platelets in a healthy individual is 150–400 × 10^9^/L of blood, with an average lifetime of 7–10 days and an average daily production of around 1 × 10^11^ platelets [2]. However, a platelet count above 400 × 10^9^/L has been observed in cancer patients [5]. The potential of tumor-educated platelets (TEPs) as a treasure trove of non-invasive biomarkers for panels of RNA cancer biomarkers that provide specific information on the presence and molecular characteristics of the disease was recently discovered [3]. The role played by platelets attracted to the TME during angiogenesis is essential for tumor growth [31].

Vascular endothelial growth factor (*VEGF*) released from α granules as a proangiogenic protein promotes vasculogenesis by stimulating endothelial migration and proliferation [32]. In the present work, we showed that this was the only gene that was differentially expressed in all the breast molecular subtypes in both types of biological samples (platelets and FFPE breast tissue). Thus, this gene may be a key factor in the development of any breast cancer subtype and could be a general biomarker for the presence of breast cancer, regardless of the molecular subtype (Figure 1a). Platelet-derived growth factor (*PDGF*) is another constituent of α-granules released upon activation of platelets that signals through its specific receptor tyrosine kinase to promote cell growth, proliferation, and differentiation [26]. This protein is another proangiogenic factor that promotes the production of extracellular matrix proteins, which directly or indirectly induce tumor angiogenesis. *PDGF* significantly influences the metastatic process through the epithelial–mesenchymal transition (EMT) process, which it promotes by activating the STAT3 or the PI3K pathway [33]. The presence of *PDGF-A* in the tumor microenvironment correlates with the invasive capacity and metastatic potential of malignant cells (as previously reported by Hadi A et al. in 2014) [34]. We found that *PDGF-A* expression was increased in platelets from the luminal A and luminal B breast cancer subtypes. In contrast, in the HER2+ and TNBC subtypes, its expression was not significantly different from the expression of *PDGF-A* in the control samples. In tumor tissue samples, the expression of *PDGF-A* was not significantly different from the expression in non-neoplastic samples (control) (Figure 1b). As expected, the expression of platelet *PDGF-A* does not depend on tumor levels. Therefore, this gene could be a marker of tumor-educated platelets in luminal A and B subtypes.

Pinto et al. [35] demonstrated in MCF7 (luminal A) cells that the expression of *PDGF* increased cell proliferation. Since they demonstrated it in vitro, they proposed that the result was due to paracrine signaling towards *PDGF* receptors present in breast cancer cells. It has been suggested by some authors that *PDGF* also plays a crucial role in therapeutic resistance. It has been shown that ER+ breast tumors of menopausal women undergoing neoadjuvant therapy with an aromatase inhibitor show an increased expression of *PDGFR-β*, which suggests that the *PDGF* pathway may be upregulated in hormone-resistant luminal tumors, making it a potential target for resistance reversal [26].

The primary function of ANG-1 is the maturation of blood vessels and the survival of endothelial cells. It has been reported that the overexpression of ANG-1 in transgenic mice induces an increase in vascularity. Moreover, while VEGF overexpression only results in increased vascular branching, VEGF-induced vessels leak [36]. In contrast, vessels induced by ANG-1 do not leak and resist leakage induced by inflammatory agents [27]. In the present work, the expression levels of *ANG-1* were found to increase in platelet and tumor samples from patients with breast cancer of the luminal A and TNBC molecular subtypes. In the HER2+ molecular subtype, *ANG-1* was found overexpressed only in tumor tissue (Figure 1c).

In 2015, Best et al. performed a massive sequencing of platelet RNA from cancer patients and healthy individuals. They found an increased expression of PF4 in cancer patients compared to platelet samples from healthy donors [17]. PF4 levels in circulation and within tumors have been associated with increased tumor growth [37]. In the present work, the expression of this gene increased in patients with luminal A breast cancer in both types of samples, platelets and tumors. In contrast, in patients with HER2+ breast cancer, the expression of *PF4* increased only in tumor tissue (Figure 1d).

Cancer is associated with changes in the organization of the actin cytoskeleton, which is related to changes in the expression levels of the different TPM isoforms [17,38]. *TPM3* is a gene that encodes the muscle protein tropomyosin alpha 3, which binds to actin filaments in muscle and non-muscle cells. TPM3 is the major isoform in cancer cells, accounting for 70% of the total tropomyosin [39]. In 2019, *TPM3* was reported in a study by Janco et al., where they characterized the gene expression signatures of platelets from breast cancer patients. They reported a high expression of TMP3 mRNA in these patients [40]. Using thromboSeq files, Yao et al. validated TPM3 by RT-qPCR as a platelet RNA marker for the detection of breast cancer, with an AUC of 0.971 on 109 healthy volunteer samples and 504 samples of patients with stage I–IV breast cancer [9]. In the present work, we found that when stratifying the population of cancer patients by molecular subtypes, *TPM3* showed variations in mRNA levels in luminal A breast cancer in both types of samples and only in platelets of TNBC (Figure 1e).

In 2021, Hinterleitner studied the expression levels of *TAC1* mRNA in breast cancer patients. In line with the present work, they observed that the expression of *TACI* in platelets derived from breast cancer patients was significantly increased compared to platelets from healthy donors (Figure 1f). According to the present results, *TAC1* showed variations in mRNA levels in platelet samples from breast cancer patients compared to tumor tissue samples. Hinterleitner concluded that the expression of *TAC1* in platelets has potential as a biomarker in liquid biopsies for breast cancer. The *TAC1* expression has been reported in several types of cancer [30]. TAC1 hypermethylation has been observed in several cancer types, including lung, colorectal, head and neck, uterine, and pancreatic cancer [41,42]. *TAC1* is a precursor gene of the tachykinin family of peptide hormones (substance P and neurokinin A) that function as vasodilators and interact with nerve receptors and smooth-muscle cells [43]. Neurokinin A inhibits cell proliferation in normal cells, so this gene is considered a tumor suppressor.

Several studies have shown that *LAPTM4B* overexpression is associated with tumorigenesis and metastatic progression in different cancers, such as hepatocellular carcinoma (HCC) [44], breast cancer [45], ovarian carcinoma [46], and gastric cancer [47]. In a study conducted in 2019, Xuelu et al. correlated *LAPTM4B* overexpression with the presence of TNBC [29]. LAPTM4B has been reported to promote cancer cell growth by activating the AKT signaling pathway [48]. In the present work, we only reported the increased expression of *LAPTM4B* in tissue samples from patients with luminal A, luminal B, and TNBC (Figure 1g).

*WASF3* is a gene that codes for a protein that forms a multiprotein complex that binds receptor kinases and actin [49]. The multiprotein complex that is generated from this union serves to transmit signals related to changes in cell shape, motility, or function, that play an essential role in cell invasion and metastasis through the activation of membrane structures [50]. Under normal conditions, these structures are formed to protect the tissues from rupture and damage. However, under pathological conditions, cancer cells take advantage of this process to invade local tissues and metastasize to other parts of the body [51]. In various types of human cancer, such as colon, prostate, and pancreatic cancer, *WASF3* is upregulated [52]. However, breast cancer is linked to increased cell-invasive and metastatic potential through the regulation of epithelial cells and EMT [28,53]. The results obtained for this gene in the present work showed variation in mRNA levels and overexpression in the luminal A and B breast cancer subtypes in both platelet and tumor tissue samples, respectively, but only variation in mRNA levels in platelets in the TNBC subtype (Figure 1h). In normal cells, *WASF3*, which is predominantly expressed in the nervous system [54], participates in the transduction of signals that lead to changes in cell morphology and cytoskeletal organization through the previously mentioned regulatory mechanism. In cancer cells, a high expression of *WASF3* correlates with more aggressive tumors [55]. In contrast, deletion of WASF3 in breast and prostate cancer cells leads to a loss of motility, invasion, and metastasis, partly related to the reduced ability to generate lamellipodia [28].

On the other hand, we showed the results of a PCA. This powerful tool allows us to differentiate between study groups of interest concerning various variables and, ultimately, relate the results to a single group with particular characteristics. In our work, the PCA showed that the *TAC1* gene could differentiate healthy samples from breast cancer samples (Figure 4). It was also observed that *VEGF* is a marker of samples with comorbidities.

In this regard, it was found, in already published works, that platelet secretion rich in proangiogenic factors has been associated with increased cell proliferation in breast cancer through *VEGF*-integrin signaling. This pathway potentiates angiogenesis, which contributes to tumor growth [56]. Interestingly, it has been documented that, in patients with DM2, circulating levels of *VEGF* are increased compared to the control group, suggesting that *VEGF* is associated with this comorbidity [57,58]. Similarly, *VEGF* has been shown to be associated with cardiovascular events and heart disease, respectively [59]. Platelet expression has great potential to be used as a reporter in the diagnosis of breast cancer by liquid biopsy, even to define the subtype of cancer against which it is intended to fight. In Figure 2, the results of differential gene expression found in each subtype of breast cancer were summarized. We observed that, as a consequence of the heterogeneity of the disease, the possible platelet markers also varied with the subtype.

It is important to note that we were not able to obtain the complete clinical history of all the patients, including their treatment scheme. This is a limitation of our work, although the importance of prior chemotherapy or radiotherapy in our results should be minor, since the samples were obtained prior to the adjuvant therapy.

## 4. Materials and Methods

**Figure 7:**
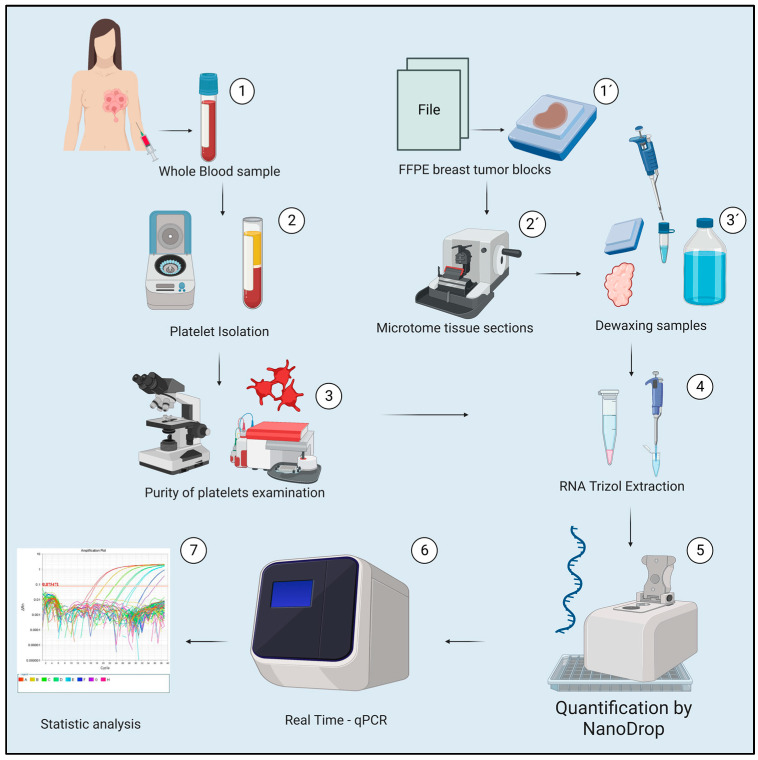
General method. (1,1′) The samples of tumor tissue and blood from different patients with breast cancer were acquired at the “Luz Cosío González” General Hospital of Zacatecas. (2,2′,3,3′) Both samples were processed by different methods, (4) Total RNA was obtained in both types of biological samples by the Trizol method and (5) quantified in Nanodrop. (6) cDNA was synthesized from a retro-transcription, quantified, and qPCR of the different genes to be analyzed in this study was performed: *PF4*, *VEGF*, *PDGF*, *ANG-1*, *WASF3*, *LAPTM4B*, *TPM3*, and TAC1. (7) The relative expression analysis was performed according to the equation of Livak and Schmittgen [60] Rn = 2^−(ΔΔCq)^, normalizing with the GAPDH gene. The corresponding statistical analysis was performed using the GraphPad Prism v. 9.0 (GraphPad Software, San Diego, CA, USA) for each study group. Created in Biorender.

Data from the diagnosis of breast cancer samples by the pathology department of the “Luz González Cosío” Zacatecas General Hospital and from the clinical record of each patient were stratified according to the general information reporting the presence of receptors (ER, PR, HER2), the breast cancer subtype (luminal A, luminal B, HER2+, triple-negative), the stage, the presence or absence of metastasis, and the presence or absence of comorbidities. The general methodology is summarized in Figure 7.

### 4.1. Ethical Statement

The research projects on platelets and FFPE breast tissue to obtain samples from donors with other breast conditions, except for cancer and from patients with breast cancer, were previously approved by the Research Ethics Committee of the Zacatecas General Hospital “Luz Cosío González”, with numbers 02043/2020 and 02222/2020, respectively. For the obtention of platelet samples, patients and healthy volunteers signed a written informed consent form to use their clinical data and donated samples in further studies. We used 10-year-old FFPE breast tissue samples kept in the archive of the Hospital’s pathology department, for which it was not necessary to obtain the signed consent of the patients. The control samples in the case of the FFPE collected came from patients with lipomas, mastitis, and fibroadenomas. All protocols were designed and performed according to the principles of the Declaration of Helsinki.

As mentioned above, the tumor tissue samples came from the General Hospital of Zacatecas files in 2012. In contrast, the whole blood samples were taken and processed in 2022—this implies that the biological samples did not come from the same patients. The disadvantage that could exist here is that we did not compare what was found in tissue and platelets from the same patients. The main advantage we see in doing it this way is evaluating possible biomarkers to diagnose the disease. Regardless of the sample’s origin, it was analyzed whether there was a relationship between the variation in mRNA levels or overexpression and the subtype of breast cancer, as well as whether or not there was a marker that had the same variation in both biological samples.

### 4.2. Whole Blood Samples

Six-milliliter blood samples were obtained from forty-four breast cancer patients and fifty healthy donors. The blood samples were collected in vacutainer tubes containing sodium citrate (3.2%) at a concentration of 0.105 M. The samples from patients were collected according to the following inclusion criteria: (1) patients diagnosed with breast cancer from the HGZ, (2) patients without aspirin treatment (3 weeks at least), and (3) patients who agreed in writing to participate in the study (signed informed consent).

The inclusion criteria for healthy volunteers were as follows: (1) not having any diagnosed disease, (2) not taking aspirin or acetylsalicylic acid* (ASA) for at least 3 weeks before sample collection, and (3) accepted in writing to participate in the study (signed informed consent).

#### 4.2.1. Platelet Purification

Whole blood extracted from patients and healthy volunteers was centrifuged at 3500 rpm for 7 min at room temperature. The resulting lower two-thirds of the plasma, consisting of platelet-rich plasma (PRP), was separated, and then concentrated by centrifugation at 4800 rpm for 7 min at room temperature. Subsequently, the platelets were washed with HEP buffer (140 mM NaCl, 2.7 mM KCl, 3.8 mM HEPES, 5 mM EGTA, pH 7.4), supplemented with 200 mM of ASA* in a 1:1 ratio to PRP. The pellet was processed by adding 500 ul of Tyrode’s buffer (134 mM NaCl, 12 mM NaHCO_3_, 2.9 mM KCl, 0.34 mM Na_2_HPO_4_, 1 mM MgCl_2_, 10 mM HEPES, pH 7.4), supplemented with BSA (0.1%) to wash away any cell debris, and then RNA extraction was performed following the Trizol method.

#### 4.2.2. Platelet Purity

The purity of the platelet isolation was determined by light microscopy using Wright’s staining. Samples were considered at optimal purity when only 0–3 nucleated cells were observed in all the fields (at least 10 were examined at 10× magnification and verified at 40×).

Note that acetylsalicylic acid (ASA), or aspirin, is an antiplatelet agent that inhibits platelet cyclooxygenase-1 (COX-1), and consequently, prevents platelet activation and any of its activities. Including an exclusion criterion for patients who consumed it three weeks prior to the sampling assured us that this factor would not interfere with the tumor-educated platelets. On the other hand, including it in the platelet isolation protocol allowed us to have platelets that did not degranulate during the process, thus ensuring that crucial genetic material was not lost and that expression patterns were not altered.

### 4.3. FFPE Breast Tumor Blocks

Fifty blocks of paraffin-embedded tissue from malignant breast tumors and fifty blocks of adjacent tissue without malignancy or tissue from patients without malignant neoplasms were obtained from the Zacatecas General Hospital “Luz Cosío González”.

#### Processing of FFPE Samples

Ten 5 µm microtome sections were obtained from each FFPE sample. The sections were placed in 1.5 mL tubes. After deparaffinization, the tissue was processed for RNA extraction following the Trizol method.

### 4.4. RNA Extraction

#### 4.4.1. RNA Extraction from Platelets

Two hundred microliters of TRIzol^TM^ Reagent (ThermoFisher Scientific, Carlsbad, CA, USA. Cat No. T-9424) was added to the total platelets previously isolated from patients and healthy volunteers, following the supplier’s protocol for extraction of total RNA from cells in suspension. The RNA concentration and purity were determined using a NanoDrop One Spectrophotometer (ThermoFisher Scientific, CA, USA). The synthesis of cDNA was then performed.

#### 4.4.2. RNA Extraction from FFPE

Here, 250 µL of proteinase K buffer (50 mM Tris pH 8.0, 400 mM NaCl, 2 mM EDTA, 4% SDS) and 50 µL of proteinase K (20 mg/mL) (ThermoFisher Scientific, CA, USA. Cat No. AM2548) were added to the previously deparaffinized samples, and the entire sample was incubated overnight at 50 °C, with shaking at 400 rpm. Subsequently, the sample was incubated for 1 h at 90 °C without shaking. Seven hundred and fifty microliters of TRIzol^TM^ Reagent were then added, and the supplier’s protocol for total RNA extraction from tissue was continued. The RNA concentration and purity were determined using a NanoDrop One Spectrophotometer. Then, we proceeded to the synthesis of cDNA.

### 4.5. cDNA Synthesis

Before synthesizing cDNA, the RNA from both platelets and tissue was subjected to a digestion process to remove contaminated DNA using DNAse I (1 U/µL) RNAse Free (ThermoFisher Scientific, CA, USA Cat No. EN0521) according to the manufacturer’s instructions.

The cDNA synthesis was performed on the purified and quantified RNA with the SuperScript™ IV Reverse Transcriptase Kit (ThermoFisher Scientific, CA, USA Cat No. 18091200). In the case of cDNA synthesis from platelet RNA, the supplier’s instructions were followed. In the case of cDNA synthesis from FFPE breast tissue samples, the protocol was as follows: incubation at 45 °C overnight, followed by incubation at 70 °C for 10 min. In both cases, oligo-random hexamer primers were used. The newly synthesized cDNA was stored at −70 °C until use.

### 4.6. qPCR Conditions

The qPCR reaction was performed in a total volume of 20 µL. The concentration of the stored cDNA was adjusted to a final cDNA concentration of 100 ng/µL. Here, 1 µL of cDNA, 10 pmol of each primer (Table 3), water, and 10 µL of the Maxima™ SYBR^®^ Green/ROX Kit mix were used (Thermo Scientific, CA, USA Cat No. K0222). The qPCR reaction was carried out in the QuantStudio 1 Real-Time PCR System (ThermoFisher Scientific, CA, USA) under the following conditions: 2 min at 50 °C, 10 min at 95 °C, followed by 40 cycles at 95 °C for 15 s, and 57.5 °C for 1 min. The samples with Ct above 39 were removed. The primers were designed at the exon–exon junction using the sequence reported for each gene in the NCBI database. The information on the primers used in the present work is presented in Table 3. The relative expression of the PF4, WASF3, LAPTM4B, ODC1, TPM3, TAC1, VEGF, PDGF, and ANG-1 genes was determined using the Livak and Schmittgen method [60], Rn = 2^−(ΔΔCq)^, normalizing with the GAPDH gene.

### 4.7. Statistical Analysis

The normality of the samples was analyzed using the Shapiro–Wilk test, and the different groups were analyzed using the Kruskal–Wallis test, considering *p*-values of * < 0.05, ** < 0.01, and *** < 0.001 as statistically significant differences. The statistical analysis was carried out using GraphPad Prism v. 9.0 (GraphPad Software, San Diego, CA, USA).

### 4.8. Principal Component Analysis

A principal component analysis (PCA) to assess the relationship between gene expression and other pathological conditions, such as comorbidities and metastasis, was performed with R software, v3.6.1 (RStudio, PBC, Boston, MA, USA) function “procomp”. The first two dimensions were chosen to describe the whole variable relationships according to their distribution.

## 5. Conclusions

Liquid biopsies are a potential non-invasive tool for the early diagnosis of cancer. They can be used to detect and monitor it, complementing or even replacing current tissue biopsy procedures. Several biosources and blood biomolecules have been explored to develop diagnostic molecular tests, such as cell-free DNA and RNA, proteins, circulating tumor cells, and extracellular vesicles. Platelets are a new potential tool for this. Disease biomarkers can be effectively evaluated by RT-qPCR, which has proven to be a powerful tool for quantitative analysis of the target mRNA expression, especially useful when there are limited biological samples, such as platelets from cancer patients.

It is vital to find biomarkers that could be used to diagnose the different subtypes of breast cancer in liquid biopsy. The main advantage is that they are non-invasive methods that can be performed at different times on a patient. It was interesting to find molecules such as TAC1 that were overexpressed in the tumor samples, although they were not overexpressed in the tumor, indicating that this was possibly associated with the tumor microenvironment rather than the tumor cells. The TAC1 gene was the one that allowed us to differentiate between a healthy sample and a breast cancer sample.

The results of the present study indicated that each of the eight genes analyzed has a different variation in mRNA levels and overexpression profiles in each of the molecular subtypes of breast cancer and biological samples. Furthermore, according to Figure 2, a molecular signature specific to each molecular subtype of breast cancer exists. However, it is necessary to increase the number of patient samples to determine the RNA(s) that differentiate different breast cancer subtypes.

On the other hand, it would be imperative to know the status of the proteins corresponding to the mRNAs with high levels in platelets, and that could directly affect the growth or metastasis of tumor cells, produce changes in the growth or proliferation of cancer cells, or modify the tumor microenvironment or prepare a premetastatic niche of cancer cells. This study is one of the next steps. However, with the current data, we can already consider some molecules as potential biomarkers for detecting or monitoring the tumor process in patients.

## Figures and Tables

**Table 1:** Characteristics of platelet samples.

Patient Characteristics Female Gender	Breast Cancer Platelets Samples *n* = 44	Healthy Donors’ Platelets Samples *n* = 50	*p*-Value *
n (%)	44 (100%)	50 (100%)	
Age (years) Mean ± SD	53.86 ± 10.96	33.86 ± 9.95	<0.0001
Clinical Pathological Data			
Classification			
Histological Grade			
0	0 (0%)		
I–II	19 (43%)		
III–IV	25 (57%)		
Receptor Expression			
Luminal A	20 (46%)		
Luminal B	8 (18%)		
HER2+	7 (16%)		
TNBC	9 (20%)		
Metastasis			
Yes	17 (39%)		
No	27 (59%)		
Comorbidities		Diagnosed diseases	
Yes	13 (41%)	9 (18%)	
Diabetes mellitus	6	2	
Hypertension	4	12	
COVID-19	-	2	
Allergies	-	1	
Colitis	1	-	
Arthritis	1	1	
Hypothyroidism	1	41 (82%)	
No	31 (59%)		
Surgical intervention			
Breast Removal	14 (31.8 %)		
Other	0 (0%)		
None	30 (68.1%)		

SD: Standard deviation; WR: without register; TNBC: triple-negative breast cancer. * Statistical analysis was realized by the Mann–Whitney test in GraphPad Prism 9.0.

**Table 2:** Characteristics of donated FFPE breast tissue samples.

Patient Characteristics	Breast Cancer FFPE Tissue *n* = 53	Healthy Donors’ FFPE Tissue *n* = 50
**Female Gender (100%)**		
n (%)	53 (100%)	50 (100%)
Age (years) Mean ± SD	55.42 ± 12.88	36.36 ± 20.11
Clinical Pathological Data		
Classification		
Histological Grade		
I	22 (44%)	
II	3 (6%)	
III	20 (40%)	
WR	8 (10%)	
Receptor Expression		
Luminal A	13 (26%)	
Luminal B	11 (%)	
HER2+	12 (%)	
TNBC	3 (12%)	
WR	14 (20%)	
Metastasis		
Yes	26 (49%)	
No	19 (36%)	
WR	8 (15%)	
Comorbidities		Diagnosed diseases
Yes	16 (31%)	7(14%)
Diabetes mellitus	8	3
Hypertension	6	4
AllergiesNo	232(60%)	-
WR	5 (9%)	43 (86%)
Surgical intervention		
Breast Removal	20 (40%)	
Other	10 (20%)	
None	20 (40%)	

SD: Standard deviation; WR: without register; TNBC: triple-negative breast cancer.

**Table 3:** Primers.

Gene	NCBI Reference Sequence	Primers	Length
ANG-1	NM_001146.5	F′-CTGAACGGTCACACAGAGAG R′-CACTGCAATGATGTTTTTCTTCG	90
VEGF	NM_001025368.3	F′-ACAAGAAAATCCCTGTGGGC R′-GCGAGTCTGTGTTTTTGCAG	98
PF4	NM_002619.4	F′-CAGCGCTGAAGCTGAAGAA R′-CCTCCAGGCTGGTGATGTG	92
PDGF	NM_002607.6	F′-GGAACGCACCGAGGAAGA R′-AGGAGGAGAAACAGGGAGTG	117
WASF3	NM_006646.6	F′-TCTGCAACCCATAGTCAACG R′-CTGGTAATCCCTTCAGGCAG	109
LAPTM4B	NM_018407.6	F′-GGAGCGATGAAGATGGTCG R′-ATCAGATACCAGACGCCGAG	107
TPM3	NM_152263.4	F′-TGCAGAAGAGGCAGATAGGA R′-CTTAGACTCTGCCAGCTCAG	106
TAC1	NM_003182.3	F′-TTGAAAAACAAGTGGCCCTG R′-TGCCCATTAGTCCAACAAAGG	96
GAPDH	NM_002046.7	F′-CACCATGGAGAAGGCTGG R′-TGCTGATGATCTTGAGGCTG	137

## Data Availability

Not applicable.

## Supplementary Materials

The following supporting information can be downloaded at: https://www.mdpi.com/article/10.3390/ijms24098348/s1.
