# Supplementary material for: Gene Expression Behavior of a Set of Genes in Platelet and Tissue Samples from Patients with Breast Cancer
Source: Int J Mol Sci. 2023 May 6;24(9):8348. doi: 10.3390/ijms24098348 (PMC10179257; doi:10.3390/ijms24098348)
Supplement: Supplementary file 1 [file ijms-24-08348-s001.zip › ijms-2342171-supplementary.pdf]

Supplementary Materials: Gene Expression Behavior of a Set of Genes in Platelet and Tissue Samples from Patients with Breast Cancer

Luis A. Burciaga-Hernandez [1,2], Cecilia F. Cueto-Villalobos [3], Nancy Ortega-Piñon [2], Irma E. Gonzalez-Curiel [4], Susana Godina-Gonzalez [5], Gwendolyne Mendez-Frausto [4], Anna P. Aguilar-Esquivel [6], Vilma Maldonado-Lagunas [7], Luis E. Guerrero-de la Torre [1,6], Jorge Melendez-Zajgla [8], Erika K. Sanchez-Garcia [7], Irma B. Mitre-Aguilar [9] and Gretel Mendoza-Almanza [1,7,10,*]

[1] Maestría en Ciencias Biomédicas, Universidad Autónoma de Zacatecas, Zacatecas 98160, Mexico
[2] Unidad Académica de Ciencias Biológicas, Universidad Autónoma de Zacatecas, Zacatecas 98068, Mexico
[3] Unidad Académica de Ciencias Químicas, Universidad Autónoma de Zacatecas, Zacatecas 98160, Mexico
[4] Laboratorio de Inmunotoxicología y Terapéutica Experimental, Unidad Académica de Ciencias Químicas, Universidad Autónoma de Zacatecas, Zacatecas 98160, Mexico
[5] Laboratorio de Biomarcadores, Unidad Académica de Ciencias Químicas, Universidad Autónoma de Zacatecas, Zacatecas 98160, Mexico
[6] Hospital General Zacatecas "Luz González Cosío", Zacatecas 98160, Mexico
[7] Laboratorio de Epigenetica, Instituto Nacional de Medicina Genomica (INMEGEN), Ciudad de México 14610, Mexico
[8] Laboratorio de Genomica Funcional del Cancer, Instituto Nacional de Medicina Genomica (INMEGEN), Ciudad de México 14610, Mexico
[9] Unidad de Bioquímica, Instituto Nacional de Ciencias Médicas y Nutrición Salvador Zubiran (INCMNSZ), Ciudad de México 14080, Mexico
[10] Consejo Nacional de Ciencia y Tecnología, Ciudad de México 03940, Mexico

* Correspondence: grmendoza@conacyt.mx

1. Age dispersion in donated samples

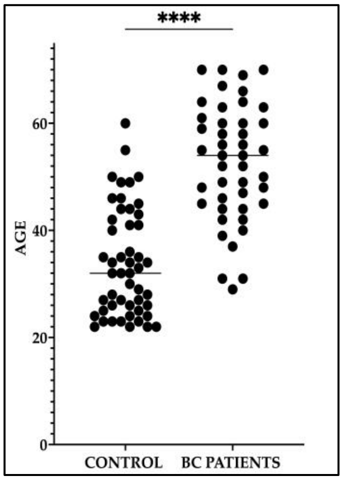

Figure S1. Age dispersion between the groups of healthy donors and patients. The age difference between the two groups from which the platelets were isolated was analyzed. The Mann-Whitney tests for statistical analysis were done in GraphPad Prism v. 9.0 (GraphPad Software, San Diego, California, USA). Asterisks represent a significant p-value ≤ 0.0001****.

2. Purity of platelets in RTqPCR assays

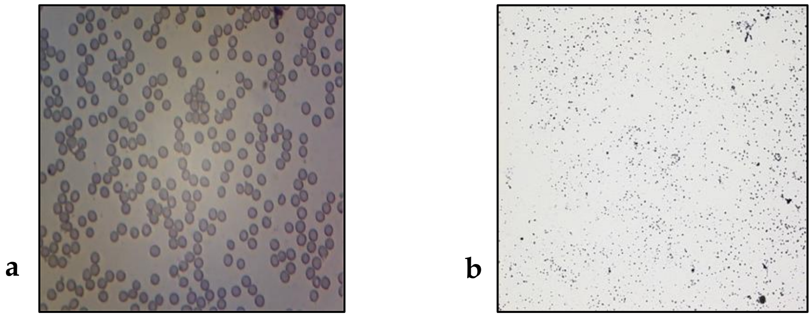

Figure S2. Confirmation of the purity of the platelet isolation by optical microscopy. The whole blood sample (a) and the isolated platelet sample (b) were stained with Wright's stain, and the samples were analyzed under an optical microscope. Figure (b) confirmed the absence of erythrocytes and leukocytes. Only the presence of platelets is appreciated. Optical microscope magnification 10X.

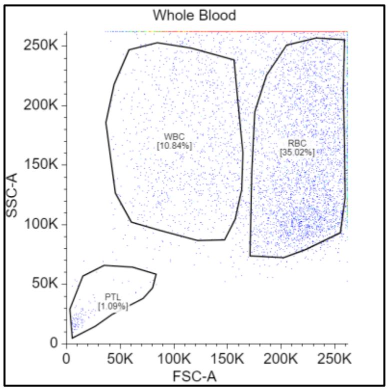

3a

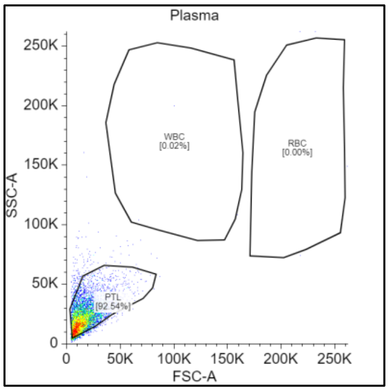

3b

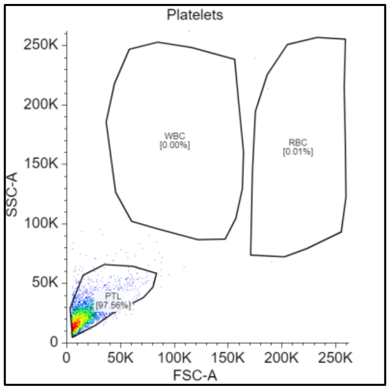

3c

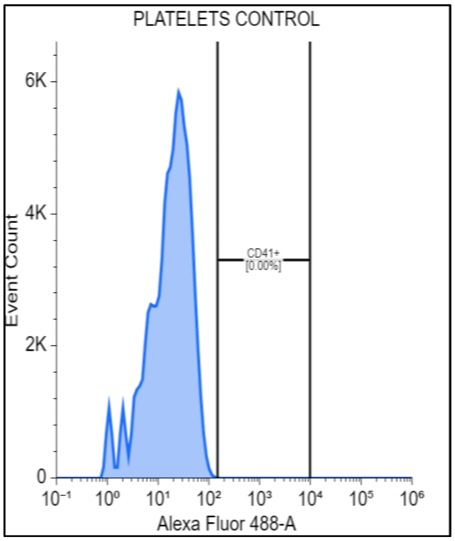

3d

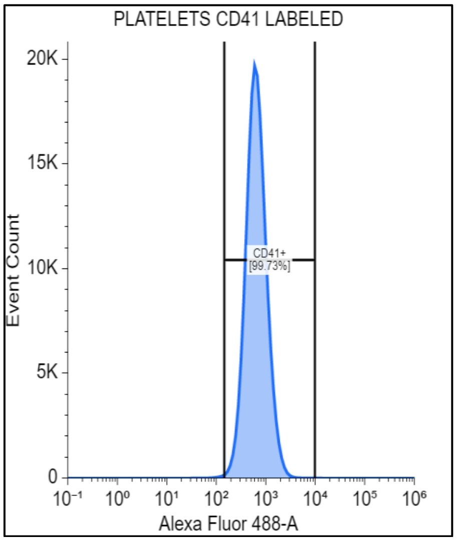

3e

Figure S3. Confirmation of the purity of the platelet isolation by Flow Cytometry. (a) The different whole-blood populations were separated according to their size. (b) After separating the platelet-rich plasma and the cell fraction by centrifugation, a second analysis in FC was performed to observe the populations that formed according to size. (c) Once the protocol of platelet purification from whole blood were finished, we performed a third analysis in FC to confirm the presence of one single cell population corresponding to platelets. (d) Once this unique population corresponding to platelets was obtained, the population was marked with CD41, and it was confirmed that it was platelets (e).

3. Impact of age on gene expression in platelets from breast cancer patients

Relative mRNA levels in patients grouped by age

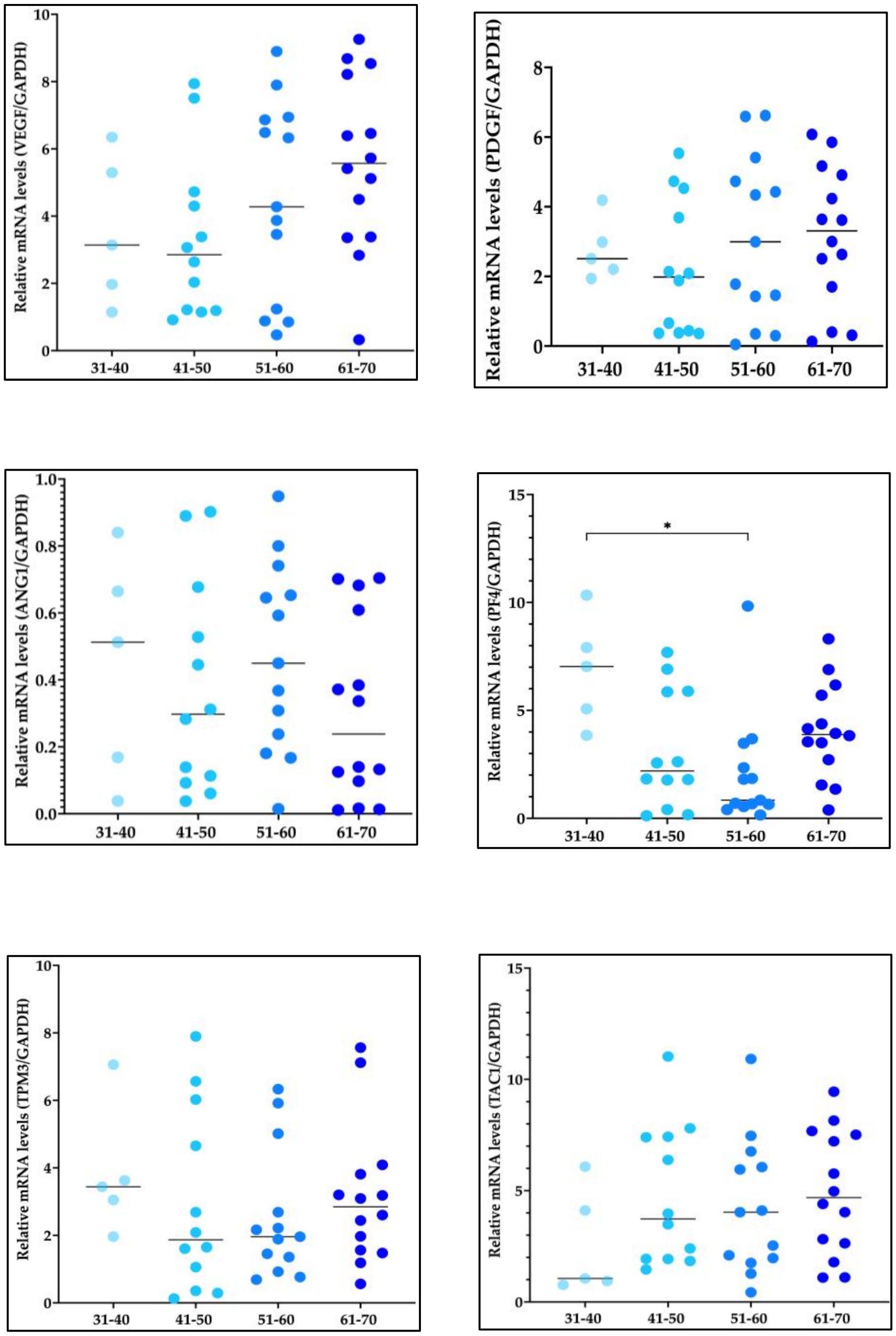

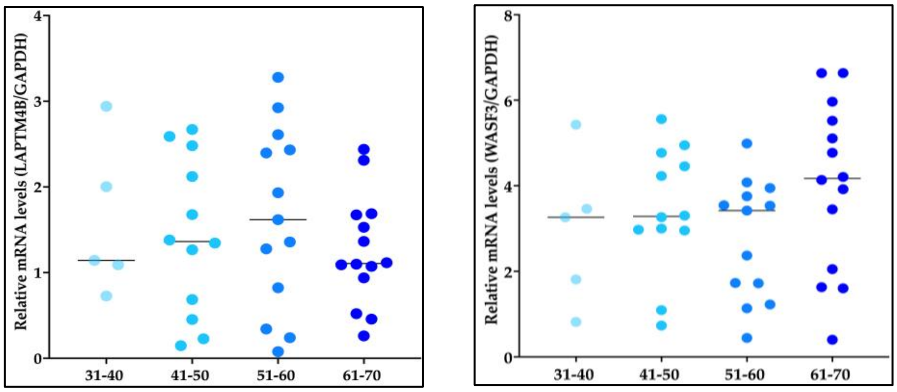

Figure 4. Impact of age on variation in mRNA levels in platelets of patients with breast cancer. Blood donor patients were grouped by age to analyze whether the changes in mRNA levels observed would be a product of the age in this group of people and not a product of their disease. According to the statistical analysis, there is no difference in gene expression between breast cancer patients related to their age. The changes depend on cancer's presence. The graphs represent the changes in the expression of the eight genes in each sample of breast cancer patients with different age ranges: patients from 31 to 40 years old; patients from 41 to 50 years old; patients from 51 to 60 years old; patients from 61 to 70 years old. Multiple comparisons were analyzed using One-way ANOVA test, considering p-values of <0.05*as statistically significant differences. The statistical analysis used GraphPad Prism v. 9.0 (GraphPad Software, San Diego, California, USA).
